# “Intravaginal eletrical stimulation for bladder training method” by Cássio L. Z. Riccetto, 2021

**DOI:** 10.1590/S1677-5538.IBJU.2021.0817

**Published:** 2022-01-10

**Authors:** Necmettin Yildiz

**Affiliations:** 1 Pamukkale University Faculty of Medicine Department of Physical Medicine and Rehabilitation Denizli Turkey Department of Physical Medicine and Rehabilitation, Pamukkale University Faculty of Medicine, Denizli, Turkey

*To the editor,*

I appreciate the interest shown for our manuscript by Dr. Cássio L. Z. Riccetto (1, 2). I support the importance you attribute to the multidisciplinary team and bladder training plus electrical stimulation in the management of idiopathic overactive bladder. The sentence “The authors concluded that the association of bladder training with intravaginal electrical stimulation was superior to isolated electrical stimulation in the treatment of overactive bladder.” is included in your editorial comment mentioning the conclusion of our study (1, 2). In order not to create a confusing situation for the readers, I wanted to state that our study result was “Bladder training added to intravaginal electrical stimulation were more effective than bladder training alone” (1).

The Author
